# Psychometric evaluation of the Estonian version of the Semi-structured Interview for Personality Functioning DSM-5 (STiP-5.1)

**DOI:** 10.1186/s40479-022-00197-7

**Published:** 2022-12-01

**Authors:** Maarja-Liisa Oitsalu, Maie Kreegipuu, Joost Hutsebaut

**Affiliations:** 1 Department of Psychology, University of Tallinn, Tallinn, Estonia; 2 North Estonia Medical Centre, Psychiatric Clinic, Tallinn, Estonia; 3 Viljandi Hospital, Psychiatric Clinic, Jämejala, Estonia; 4 Viersprong Institute for Studies On Personality Disorders, Halsteren, The Netherlands; 5 Department of Medical and Clinical Psychology, Tilburg University, Tilburg, The Netherlands

**Keywords:** Personality assessment, Personality functioning, Semi-structured interview STiP-5.1

## Abstract

**Background:**

The DSM-5 Alternative Model for Personality Disorders introduced a dimensional perspective on personality disorders. The model assesses functioning in four domains: Identity, Self-Direction, Empathy, and Intimacy. This study evaluates the psychometric properties of the Semi-Structured Interview for Personality Functioning DSM-5 (STiP-5.1) in Estonian.

**Method:**

The sample consists of 131 participants: 58 from the general population and 73 from a mixed clinical sample that is further divided into a mood and anxiety disorder sample and personality disorder sample. All participants completed the STiP-5.1 interview and the Level of Personality Functioning Scale–Brief Form (LPFS-BF 2.0).

**Results:**

The Estonian STiP-5.1 interview has good internal consistency (McDonald's ω between .94–.98) and high convergent validity (correlations with LPFS-BF 2.0 above .7). Interview scores successfully differentiated the general population from the mixed clinical sample (Cohen’s *d* = 2.68), as well as patients with personality disorder from those without (Cohen’s *d* = 1.76). The LPFS-BF 2.0 total score differentiates the general population sample from the mixed clinical sample (Cohen’s *d* = 1.99) but not the personality disorder sample from other clinical sample participants.

**Conclusions:**

The properties of the Estonian STiP-5.1 replicate those of other languages, and empirically support a unified personality functioning dimension that can be meaningfully thought of as reflecting impairments in self and interpersonal functioning. Findings of this study will be discussed in the light of the ongoing debate on the dimensionality of personality pathology and the use of self-report versus interview measures for assessing personality pathology.

## Introduction

In the last two decades, personality disorder diagnoses have slowly but steadily moved away from its traditional categorical system towards a more empirical dimensional model. The trend was evident in the Alternative Model for the DSM-5 [1], and even more so in the ICD-11 [25] in which a dimensional model of personality dysfunction is the main basis for assigning personality disorder diagnoses.

The two diagnostic classifications are fairly similar when it comes to how personality dysfunction is conceptualized. Both define dysfunction as impairments in self and interpersonal functioning that can be classified according to severity, either mild, moderate or severe. Once severity has been assessed, the clinician has the option of assessing maladaptive personality traits that contribute to the expression of personality dysfunction. These traits are based on the prevalent Big-5 trait theory, and have been proposed to reflect the pathological extremes of these five basic personality traits [26]. Although DSM-5 and ICD-11 have included slightly different traits in their systems, both count five, and agree upon negative affectivity, detachment, dissociality and disinhibition as being important for describing the different ways personality dysfunction can be expressed. Combinations of personality dysfunction and traits have successfully been mapped onto the existing categorical diagnoses as well [8].

The rationale for moving towards dimensional assessment has been to reduce diagnostic overlap, provide a better fit for empirical data as well as better inform clinical decision making and simplify the diagnostic process [4, 6]. Early data indicated that generalized severity is the single most important predictor of current and future pathology [16, 22]. This would mean that in order to make a clinically informative diagnostic decision, the clinician would not have to spend large amounts of time and resources assessing all specific aspects of functioning or establishing the nuances of specific diagnostic criteria. Early on doubts were raised about the amount of information, training and experience needed to make a reliable assessment of such general personality functioning using interviews available at the time [18, 28]. Interview data demonstrated that inexperienced assessors took a long time, and produced acceptable, yet not ideal interrater agreement [28].

There are several self-report scales for assessing personality functioning according to DSM-5 [12, 13, 17, 21, 24] and ICD-11 [3, 11]. The American Psychiatric Association has developed a diagnostic interview assessing personality functioning—the Structured Clinical Interview for the DSM-5 Alternative Model for Personality Disorders Module I (SCID-5-AMPD-I, [10]). An interview measure for the ICD-11 is currently still being constructed [2].

One of the first interviews specifically designed for assessing severity levels of personality functioning was the Semi-structured interview for Personality functioning DSM5 (STiP-5.1), aimed to be brief and reliable for use after only brief training. The clinician rated interview contains 60 descriptors of severity but has an integrated ‘funnel’ structure to narrow down possible levels of impairment instead of going through them all. There are four sections (identity, self-direction, empathy, intimacy), each beginning with an open question, and followed by optional additional questions that help to narrow down possible levels of impairment. The instrument has good to excellent interrater reliability, high internal consistency and construct validity [18]. Since then the STiP-5.1 interview has been translated into other languages (e.g. English, Czech, German), in each it has demonstrated good psychometric properties as well as time-efficiency taking on average as little as 38 min to administer for the German version [27]. The Czech version demonstrates that the results can be interpreted well both looking at the total score of personality dysfunction, as well as viewing the self and interpersonal dysfunction as separate facets of personality dysfunction [15]. Currently it is a well-accepted and recommended instrument in the good clinical practice of personality assessment that works for both the DSM5 as well as the ICD-11 classification systems [2, 23].

## Method

### Participants

The sample consists of 131 individuals, 58 of whom belong to the general population sample, and 73 to a mixed clinical sample. Within the mixed clinical sample, 38 patients have an ICD-10 diagnosis of a mood and/or anxiety disorder, and 28 patients have an ICD-10 diagnosis of a personality disorder. Within the mood/anxiety disorder group the majority of participants have the ICD-10 diagnosis of either major depressive disorder, single episode, moderate (F32.1; 10 participants, 26%); mixed anxiety and depressive disorder (F41.2; 10 participants, 26%); or recurrent depressive disorder, moderate, without psychotic symptoms (F33.2; 9 participants, 24%). Within the personality disorder group 13 patients (46%) have a diagnosis of personality disorder not specified (F60.9), 12 (43%) have a diagnosis of borderline personality disorder (F60.31), and 3 (11%) have the diagnosis of mixed type personality disorder (F61). Diagnoses were assigned by their treating psychiatrist based on clinical assessment.

Participants' ages range from 15 to 75, 19% of participants are 19 years or younger, 20% are between 20–25 years old, 19% between 26–30 years old, 21% between 31–39 years old, 9% between 40–49 years old, and 12% over the age of 50. In the total sample, 17% of the participants have obtained primary education, 16% secondary education, 8% vocational education, and 28% have a university degree.

There are slight demographic differences between the general population and the mixed clinical population. The ratio of male participants in the general population sample is 57% as compared to 26% male in the clinical sample.

There are differences in education, with 36% of the general population sample having obtained a university degree, compared to 22% in the clinical sample. Furthermore, 29% of the clinical sample has primary education as their highest attained education, compared to 3% in the general population. This is at least partially due to the fact that the clinical sample is significantly younger than the general population sample. 73% of the clinical sample is younger than 30 years old, as compared to 41% under 30 in the general population.

The sample demographic differences reflect real life differences, at least to a certain degree. Research indicates that females tend to seek more medical care than males [20], and symptoms of personality dysfunction are often most prevalent and disturbing in young adulthood, often subsiding in their natural course with age [14].

Participants in both the general population and patient sample were recruited using snowball methodology by the participants who took part in the STiP-5.1 interview training. Patient status was not recorded—the sample includes both inpatient and outpatient participants.

### Instruments

#### Semi-structured interview for Personality functioning DSM-5 (STiP-5.1)

The STiP-5.1 [18] is a clinician-rated semi-structured interview for the assessment of overall personality functioning. The interview results in a general personality functioning score and two main domains of self and interpersonal functioning that each have two elements: self functioning consists of identity and self-direction, and interpersonal functioning of empathy and intimacy. Clinicians rate all of these aspects of functioning on a scale of 0 (no impairment) to 4 (extreme impairment). The administration takes on average 50 min in the original version, ranging from 28 to 70 min.

Previous versions of the STiP-5.1 have shown good internal consistency with Cronbach ɑ of 0.97 for the original scale, and interrater reliability of ICC = 0.93 for the German version.

The interviewers in this study were either trained by the author of the original version (J. Hutsebaut) during a 1 day workshop or trained by participants of this workshop who had used the STiP-5.1 in clinical practice for more than 2 years. The latter training included 4 times 4 h workshops where theoretical background of the instrument was given, and the interview process and scoring was practiced. The study interviews were conducted after the participants had practiced carrying out the interview under supervision and had scored an interview example. The interview length in this study was generally 60 min or more, since the participants were encouraged to ask for examples and use the additional questions to improve their scoring accuracy.

#### The Level of Personality Functioning Scale–Brief Form (LPFS-BF 2.0)

The LPFS-BF 2.0 [7] is a brief self-report version of the original Level of Personality Functioning scale [9]. The LPFS–BF was initially constructed and empirically evaluated in Dutch, and subsequently translated to English [19]. The scale consists of 12 items related to dysfunction in identity, self-direction, empathy and intimacy, each rated on a 4-point Likert scale (from 0 = *very false or often false* to 3 = *very true or often true*). The scale results in one global dimension score that can be interpreted as overall personality dysfunction.

With the author’s permission the scale was translated into the Estonian language, and tested out in both general population and patient samples with participants being able to give feedback on scale items. It was then overviewed by two clinical psychologists, and translated back into the English language to ensure sufficient similarity to the original scale.

The preliminary Estonian version of the LPFS demonstrates adequate psychometric properties. Exploratory factor analysis suggests a one factor solution, with confirmatory factor analysis fit indices for this model being χ^2^(54) = 137, *p* < .001; CFI = .83, TLI = .70, RMSEA = .13, SRMR = .09. Fit indices are in the same range, if slightly higher for the two factor model (χ^2^(53) = 107, *p* < .001; CFI = .89, TLI = .86, RMSEA = .11, SRMR = .07), resulting in two interpretable subscales of self and interpersonal functioning, similar to the original version [19]. Total scale reliability is high with McDonald’s ω = .91, and the subscales are also internally consistent: Self functioning ω = .88, and Interpersonal functioning ω = .82.

## Results

### Factor Structure of STiP-5.1

Exploratory factor analysis (parallel analysis, principal components, promax rotation) suggested a two factor solution. The first factor contains the self functioning items with factor loadings between .60 and .95, and the second factor contains all the interpersonal functioning items with factor loadings between .56 and 1.01. The factors are strongly correlated (.78). Results of the exploratory factor analysis are presented in Table 1.

**Table 1:** Factor structure of the Estonian STiP-5.1

**STiP5.1 Facets**	**Factor 1** **Self functioning**	**Factor 2** **Interpersonal functioning**
STiP5.1_2 Self- direction	.95	
STiP5.1_2.1 Goals	.94	
STiP5.1_1 Identity	.88	
STiP5.1_Self functioning	.83	
STiP5.1_1.2 Self- esteem	.81	
STiP5.1_ 1.3 Emotion regulation	.81	
STiP5.1_2.2 Standards	.80	
STiP5.1_1.1_Unique self	.62	
STiP5.1_2.3 Self-reflection	.59	
STiP5.1_3 Empathy		1.01
STiP5.1_Interpersonal functioning		.89
STiP5.1_4.3 Mutuality		.87
STiP5.1_3.2 Perspectives		.87
STiP5.1_3.1 Comprehension of others		.81
STiP5.1_4 Intimacy		.72
STiP5.1_3.3 Impact		.66
STiP5.1_4.1 Connections		.58
STiP5.1_4.2 Closeness		.58

Parallel analysis with promax rotation

*Abbreviations*: *STiP-5.1* The Semi-Structured Interview for Personality Functioning DSM–5

Confirmatory factor analysis was carried out for the unidimensional as well as the two-dimensional solutions. The unidimensional model fit indices are χ^2(^135) = 975, *p* < .001; CFI = .75, TLI = .71, RMSEA = .23, SRMR = .06, GFI = .47. The two-dimensional model fit indices are χ^2^(134) = 647, *p* < 0.001; CFI = .85, TLI = .82, RMSEA = .18, SRMR = .04, GFI = 0.63. The results indicate that neither the one nor two-dimensional model is an optimal fit for the data, however the two dimensional model fits better. The fit indices are similar to the ones reported for the Czech version of the STiP-5.1 [15]. Given the sample size, and the fact that the fit indices have improved as the sample grew, it’s reasonable to expect that both the one-dimensional factor reflecting overall personality functioning, as well as the two factor solution separating self and interpersonal functioning are reasonable descriptive models for clinical use.

### Reliability of STiP-5.1 scales

For all scales, McDonald’s ω was calculated to assess the scales’ reliability. The Overall functioning scale shows very good internal consistency ω = .98, *p* < .001, as do the facets of Self (ω = .94, *p* < .001) and Interpersonal functioning (ω = .96, *p* < .001). The reliability values for all scales are presented in Table 2. There were no items for any facets reported that would, when removed, increase the scale’s reliability.

**Table 2:** STiP-5.1 scale reliability and differences in sample scores

**STiP5.1 Facet**	**Total sample**		**General population**			**Difference**^**a**^	**Mood and anxiety sample**			**Personality disorder sample**			**Sample differences**
	**McDonald's ω**	**ICC coefficient**	**Range**	**Median**	**Mean(*SD*)**	***Cohen’s d***	**Range**	**Median**	**Mean(*SD*)**	**Range**	**Median**	**Mean(*SD*)**	***Cohen’s d***
STiP-5.1 Overall	.98	.93	0–1	0	0.11(0.32)	2.68	0–3	1	1.40(0.82)	2-3	3	2.60(0.50)	1.76
STiP-5.1 Self	.94	.95	0–1	0	0.14(0.35)	2.97	0–3	2	1.6(0.85)	2-4	3	2.71(0.55)	1.55
STiP-5.1 Interpersonal	.96	1. 00	0–2	0	0.19(0.45)	1.9	0–3	1	1.1(0.85)	1-4	2	2.42(0.72)	1.68
STiP-5.1 Identity	.92	.95	0–1	0	0.33(0.47)	2.73	0–4	2	1.74(0.88)	2-4	3	2.80(0.51)	1.47
STiP-5.1 Self-direction	.93	.95	0–1	0	0.14(0.35)	2.19	0–3	1	1.26(0.91)	0–3	2	2.25(0.74)	1.19
STiP-5.1 Empathy	.94	.95	0–2	0	0.27(0.53)	1.44	0–3	1	0.85(0.84)	0–4	2	2.08(0.93)	1.39
STiP-5.1 Intimacy	.95	1	0–1	0	0.21(0.41)	1.95	0–3	1	1.28(1.03)	0–3	3	2.42(0.83)	1.22

*Abbreviations: STiP-5.1 *the Semi-Structured Interview for Personality Functioning DSM–5

^a^ Difference statistics are calculated between General population and Mixed clinical sample as a whole

For interrater reliability, a random sample of 10 interviews was selected from the mixed clinical sample. All interviews were assessed by two raters—the interviewer and an observer rating the taped interview. No interview was assessed by the same two raters in order to minimize a possible rater effect. Between rater agreement was very good for all the scales, ranging from ICC coefficient .93 to 1.00. Values for each facet are presented in Table 2.

### Age and gender differences

There were no significant gender differences in the total sample or for the mixed clinical and general population group on the STiP-5.1 Overall functioning and Interpersonal functioning scores. A small gender difference effect was observed in the mixed clinical sample for the Self functioning score (*U* = 524, *r*_rb_ = -.35, *p* < 0.001, Cohen’s *d* = -0.65), with women having slightly higher scores (*M* = 1.55, *SD* = 1.18) than men (*M* = 0.81, *SD* = 1.08) indicating more problematic functioning in this element.

No significant age differences were present in the STiP-5.1 Overall functioning and Interpersonal functioning scores. However, in the mixed clinical sample there was a small age difference in the Self functioning score, with younger age being associated with higher dysfunction (*F*(4) = 3.98, *p* < .001, ω = .15; *⍴* = -.39, *p* < .001, Cohen’s *d* = 0.7). In the general population sample gender and age had no significant correlations with any facets of personality dysfunction.

### Discriminative validity

The mixed clinical sample differed significantly from the general population on the STiP-5.1 Overall functioning score (*U* = 190, *p* < .001, *r*_rb_ = -.89; Cohen’s *d* = 2.03), as well as both on the Self functioning (*U* = 132, *p* < .001, *r*_rb_ = -.93; Cohen’s *d* = 2.97) and Interpersonal functioning scores (*U* = 435, *p* < 0.001, *r*_rb_ = -0.77; Cohen’s *d* = 1.90). Furthermore, the Overall functioning score differs significantly within the mixed clinical sample for those with personality disorder diagnoses vs those with mood and anxiety disorders (*U* = 98, *p* < .001, *r*_rb_ = -.74; Cohen’s *d* = 1.76). The same is true for both the Self functioning (*U* = 153, *p* < .001, *r*_rb_ = -0.67; Cohen’s *d* = 1.55) as well as the Interpersonal functioning scores (*U* = 128, *p* < .001, *r*_rb_ = -.73; Cohen’s *d* = 1.68). Details for these group differences are presented in Table 2.

The STIP-5.1 overall scores range from 0–1 in the general population sample, and from 2–3 in the personality disorder diagnosis sample, which is in line with the theoretical assumption that the score 2 marks the level where dysfunction characteristic of personality disorders starts [18]. The overall scores in the mood and anxiety disorder sample range from 0 to 3 meaning that at least some participants (14 participants, 37%) in that group have personality dysfunction similar to the extent of people with personality disorders, and some (6 participants, 16%) have normal personality functioning, while half fall in between in the problematic functioning level. Detailed score ranges are presented in Table 2.

The mixed clinical sample differed significantly from the general population on the LPFS-BF2.0 (*U* = 180, *p* < .001, *r*_rb_ = -.80; Cohen’s *d* = 1.99) total scores. The LPFS-BF2.0 total score was not able to differentiate the personality disorder group from the anxiety and mood disorders group within the clinical sample.

### Convergent validity

Both STiP-5.1 and LPFS-BF2.0 provide an overall personality functioning score, and these scores have been correlated using Spearman’s ⍴ to indicate convergent validity. Correlations between these overall scores are moderately strong (*⍴* = 0.77, *p* < 0.001), as are correlations between STiP-5.1 Self and Interpersonal functioning scores with the respective LPFS-BF subscales. All correlations are reported in Table 3.

**Table 3:** Spearman's correlations for STiP-5.1, LPFS and PDS-ICD11

	**STiP-5.1 Overall**	**LPFS-BF Total**	**LPFS-BF** **Self**	**LPFS-BF** **Interpersonal**
STiP-5.1 Self	.95**	.76**	.73**	.68**
STiP-5.1 Interpersonal	.86**	.72**	.63**	.73**
LPFS-BF Total	.77**			
LPFS-BF Self	.71**	.91**		
LPFS-BF Interpersonal	.72**	.89**	.67**	

*Abbreviations*: *LPFS-BF* The Level of Personality Functioning Scale–Brief Form, *STiP-5.1* The Semi-Structured Interview for Personality Functioning DSM–5

** p < .001

## Discussion

With the increasing use of dimensional personality disorder models as well as in accordance with recent European recommendations for personality assessment as outlined by [23], there was a need for an assessment instrument to measure personality functioning. There are a few patient report scales constructed for the ICD-11 [3, 11],however, since an interview instrument specifically created for the ICD-11 is still under construction [2], the Semi Structured Interview for Personality Functioning DSM5 (STiP-5.1) was chosen for these purposes. The psychometric properties of its Estonian version were evaluated in this study, alongside with a short self report instrument for screening personality functioning.

Our results support a two factor structure for the STiP-5.1 data, similar to the Czech version [15]. The resulting Self functioning and Interpersonal functioning scales have high internal consistencies and are strongly correlated with their respective scales in the Levels of Personality Functioning brief form (LPFS-BF), also best described by a two factor structure. This adds to the increasing empirical evidence that personality functioning can meaningfully be thought of as consisting of self and interpersonal functioning. The reliability indices are similar to other versions of the STiP-5.1 in both the scales’ internal consistencies as well as interrater agreement [18, 27]. Our interrater agreement is good, and even exceptionally high on some scales, which might be due to the fact that our raters had more training than has been reported in other studies and carried out longer interviews, thus possibly containing more information to score reliably.

The STiP-5.1 Self and Interpersonal functioning scales are also highly correlated, as well as strongly correlated with the LPFS-BF2.0 total score, providing support for an Overall personality functioning score as a useful and empirically sound concept. The one-dimensional structure for both the STiP-5.1 as well as the LPFS-BF is only slightly less optimal than the two-dimensional one as indicated by our confirmatory factor analysis results.

All three facets of the STiP-5.1 interview—Overall, Self and Interpersonal functioning—are significantly different between the general population and the mixed clinical sample, indicating good discriminative validity. What is more, the scales allow differentiation inside the mixed clinical sample between those patients with an ICD-10 personality disorder diagnosis and those without, presenting with mood and/or anxiety disorder symptoms. The score ranges between those groups are overlapping, however median scores for all facets are higher in the personality disorder group as compared to the mood/anxiety disorder group. The median overall functioning score in the personality disorder group is 3, remaining above the cutoff for personality disorder that is considered to be a score of 2, and there are no median scores falling below that threshold in the group. At the same time, there are also median scores of 2 in the mood/anxiety group for the Self functioning element, and its Identity aspect as well. Higher self and identity dysfunction might reflect the fact that our clinical population was very young thus allowing for more age related issues with finding one's identity, as well as possible undiagnosed personality disorders due to age and/or short period of observation. Some of this might be explained by the fact that our clinical sample was overwhelmingly female, as females often report more symptoms, and reported more self dysfunction in the clinical sample in this study as well. These small age and gender differences were also present in the Czech study [15].

On the other hand, higher prevalence of more severe self dysfunction among those patients diagnosed with mood and anxiety disorders might point us towards the fact that problems in interpersonal functioning could be more specific to personality disorders, whereas dysfunction in self functioning might more easily accompany other disorders as well.

Furthermore, this overlapping of scores is well in accordance with the dimensional model of personality functioning, where dysfunction should increase gradually, and cutoff scores are mainly imposed for clinical usefulness and do not in themselves reflect any qualitative differences in disorder [2].

The total score of the LPFS-BF2.0 is strongly correlated with STiP-5.1 Overall functioning score, as well as the Self and Interpersonal functioning scores, and was able to discriminate between the general population and mixed clinical sample, indicating that it is an adequate screening method for identifying personality problems. However, these self-reported total scores were not able to differentiate patients with personality disorders within the mixed clinical sample, making it clear that a more thorough assessment is needed to identify dysfunction specifically related to personality disorder. This is again in accordance with current clinical suggestions that for the assessment of personality disorder, self-report instruments are not sufficient [23].

## Clinical implications

The STiP-5.1 is a useful instrument for collecting precise and clinically informative data regarding personality functioning that assists in distinguishing personality disorder from dysfunction related to other psychiatric illnesses. However, precise scoring is made difficult by interviewers having to rely on a patient's introspective capacities, as well as there not being an overarching principle for assigning scores throughout the instrument. The different facets draw on different theoretical backgrounds; and several are quite abstract in their nature which means precise scoring is difficult, as well as time consuming, echoing concerns expressed by Zimmermann [28].

To cope with these issues of reliable and precise scoring, our interviewers often resorted to the use of most all questions in the STiP-5.1, as well as relevant patient examples, resulting in longer interview times. It was also evident that clinicians more experienced with the instrument and personality assessment were able to carry the interviews out in a shorter time frame but most of the interviews lasted over 60 min, which is longer than reported for the original and the German version [18, 27] that had interviews about 50 and 38 min long on average, but more similar to the Czech version [15] where interview length of 45–70 min was reported. Interview length was not specifically measured or standardized in our study, so this could possibly be an avenue for future research.

The ICD-11 has focused on finding common ground between the DSM5 and the new ICD-11 personality disorder classifications, and there have been several studies comparing the two, finding more common ground than differences [5, 6]. Following from this it should be reasonable to expect the STiP5.1 to be a useful instrument for the assessment of ICD-11 personality disorders. Furthermore, as seen in our study, higher STiP-5.1 scores are also related to ICD-10 personality disorder diagnoses, indicating that the instrument is capable of assessing facets of personality dysfunction across classification systems. The STiP-5.1 interview as well as the LPFS-BF2.0 scale both assess core personality functioning aspects such as self and interpersonal functioning but not other aspects of personality disorder, such as cognitive or behavioral manifestations, and overall psychosocial functioning. Following this, it would be expected that for a comprehensive assessment additional instruments and points of information are necessary.

## Limitations

One of the main limitations of this study was that the interview times were not recorded as separate variables. The interview time and the procedure of how thoroughly it is carried out might significantly influence interrater reliability and carry important clinical implications, and thus should be an area of further studies. Our sample was somewhat small, and the clinical sample was significantly younger than the general population sample, which reflects real life differences in these demographics one the one hand, but at the same time does influence the extent to which conclusions can be drawn about personality functioning as a general concept based on these results.

## Conclusions

Overall, this study demonstrates good psychometric properties for the Semi-structured Interview for Personality Functioning DSM5 (STiP-5.1) in the Estonian language and adds to the knowledge base that it is a reliable and valid instrument for assessing personality dysfunction. Our data demonstrate that personality functioning can be thought of as a unified dimension that can meaningfully be separated into self and interpersonal functioning. Both the STiP-5.1 interview as well as the self report scale LPFS2.0 can reliably distinguish between the general population and the mixed clinical sample, whereas only the interview was able to also reliably distinguish the personality disorder sample within the mixed clinical sample. This is an important result indicating a self-report measure alone is not reliable as a solo means for assessing personality disorder. Furthermore, it also demonstrates that the STiP-5.1 interview is a suitable instrument for assessing personality dysfunction across classification systems.

## Data Availability

The datasets used in the current study are available from the corresponding author upon reasonable request.
